# Validity, Reliability, and Diagnostic Cut-off of the Kinyarwandan Version of the Hamilton Depression Rating Scale in Rwanda

**DOI:** 10.3389/fpsyg.2020.01343

**Published:** 2020-07-03

**Authors:** Peter Dedeken, Joao Ricardo Nickenig Vissoci, Fidele Sebera, Paul A. J. M. Boon, Eugene Rutembesa, Dirk E. Teuwen

**Affiliations:** ^1^Department of Neurology, Heilig Hart Hospital, Lier, Belgium; ^2^Corporate Societal Responsibility, UCB Pharma, Brussels, Belgium; ^3^Department of Neurology, Ghent University Hospital, Ghent, Belgium; ^4^Division of Emergency Medicine, Department of Surgery, Duke Global Health Institute, Duke University, Durham, NC, United States; ^5^Department of Neurology, Neuropsychiatric Hospital, CARAES Ndera, Kigali, Rwanda; ^6^Department of Neurology and Psychiatry, University of Rwanda, Kigali, Rwanda

**Keywords:** Rwanda, Hamilton Depression Rating Scale, diagnosis, depression, cut-off, validation

## Abstract

**Introduction**: In Rwanda, major depressive disorder affects 11.9% of the population and up to 35% of genocide survivors. Mental health services remain underutilized due to stigma and lack of awareness. Increasing the ability and capacity to diagnose and treat mental disorders is considered important to close this gap. We describe the translation, validity, and reliability assessment of the Hamilton Depression Rating Scale (HDRS) as a diagnostic tool for moderate to severe depression in Rwanda.

**Methods**: The HDRS-21 was translated by a multi-group taskforce. We validated the translation against expert assessment in a comparative study on a sample of patients living with depression and of healthy volunteers. Psychometric properties, namely internal structure, reliability, and external validity were assessed using confirmatory factor analysis, three reliability calculations, and correlation analysis, respectively. Maximized Youden’s index was used for determining diagnostic cut-off.

**Results**: The translated version demonstrated a kappa of 0.93. We enrolled 105 healthy volunteers and 105 patients with confirmed mild to severe depression. In the confirmatory factor analysis, HDRS had good factor loadings of 0.32–0.80. Reliability coefficients above 0.92 indicated strong internal consistency. External validity was shown by good sensitivity (0.95) and specificity (0.94) to differentiate depression from absence of depression. At a cut-off point of 17 for the diagnosis of depression, sensitivity and specificity were both 0.95 relative to gold standard.

**Conclusion**: The validated HDRS in Kinyarwanda with diagnostic cut-off provides mental healthcare staff with an accurate tool to diagnose moderate to severe depression, enabling closure of the diagnosis and treatment gap.

## Introduction

Depression is consistently ranked as one of the most important contributors to disability worldwide, even the single largest contributor to global disability in 2015 (World Health Organization, 2017). The global prevalence of depression is estimated to be 4.4%, with the highest at 5.9% reported among women in the African region (World Health Organization, 2017). In Rwanda, with nearly 13 million citizens (Worldometer, 2020), specifically, depressive disorders were the third cause of disability (Institute for Health Metrics and Evaluation, 2019). The burden of mental disorders is compounded by the continuing impact of the 1994 genocide (Munyandamutsa et al., 2012; Rugema et al., 2013, 2015; Ng and Harerimana, 2016), not surprisingly, as conflict causes extensive mental issues (Lafta, 2016).

Mental health care in Rwanda has a pyramidal structure, ensuring decentralized care (GOV.UK, 2017; Ministry of Health Republic of Rwanda, 2018) in all public health facilities, ranging from primary health centers under supervision of secondary district hospitals to tertiary reference hospitals. In 2016, more than 250,000 mental health consultations were performed, distributed over primary to tertiary centers. Depression accounted only for 4.7% of all visits (Ministry of Health Republic of Rwanda, 2018).

In a large-scale survey conducted in 2008 with the support of the Rwandan Ministry of Health, the prevalence of post-traumatic stress disorder and major depressive episodes were 26.1 and 22.7%, respectively (Munyandamutsa et al., 2012).

In a recent survey using the Kinyarwandan version of the Mini International Neuropsychiatric Interview, it was found that major depressive disorder affected 11.9% of the general population in Rwanda, with a higher prevalence in persons aged >46 years and with lower education. Major depressive disorder amounted to 35% among genocide survivors. Despite this high prevalence, significant proportions of the population either are not aware of the availability of mental health support or do not use the available services. While 62% of the population was aware of places offering mental health support, only 5.3% used mental health services, resulting in a large diagnosis and treatment gap. Among the recommended measures to improve mental health, increasing the ability and capacity of health personnel to assess and treat early symptoms of mental disorders was considered important (Rwandan Mental Health survey 2018, unpublished).

Enabling healthcare professionals (HCPs) to identify and diagnose depression is an important first step in mental health care. In a resource-limited environment with only few specialized HCPs, a broad use of scales to screen, diagnose, and follow-up depression can contribute to closure of the diagnosis and treatment gap (Anderson et al., 2002). The Hamilton Depression Rating Scale (HDRS) is a widely used tool to diagnose and rate the severity of depression (Williams, 2001; Ruhe et al., 2005). The HDRS-21 is composed of 17 diagnostic questions, of which eight are scored on a 5-point scale, ranging from 0 to 4 and nine are scored from 0 to 2. Questions 18–21 further qualify the depression (Medscape). The total HDRS score is calculated as the sum of the first 17 questions, ranging from 0 to 52. A score of 7 or less is considered as absence of depression, whereas it is generally agreed that a score of 17–23 represents moderate depression (Zimmerman et al., 2013). It has been used extensively for assessing severity of depression, changes over time, and treatment efficacy with high levels of internal consistency, inter-rater reliability, and test-retest reliability (Williams, 2001; Ruhe et al., 2005; Trajkovic et al., 2011).

We describe the translation process of the HDRS-21 for use in Rwanda. In many African societies, it is not culturally appropriate to ask directly about emotional states such as “Have you been depressed?” and careful wording is required. Also, words describing feeling states are sometimes totally absent in the indigenous language (Mbewe et al., 2013). Subsequently, we evaluated its psychometric properties in terms of internal structure, reliability, and cut-offs for diagnosis of moderate to severe depression in a Rwandan population.

## Materials and Methods

### Translation of HDRS-21

The HDRS-21 was translated from French into Kinyarwanda by two groups of experts, who are fluent in French, English, and Kinyarwanda and are familiar with the HDRS questionnaire: one group of three clinical psychologists and one group of psychiatrists and psychiatric nurses. First, each group translated the HDRS from French to Kinyarwanda. After comparison of these two versions, an initial single translation after consensus was proposed. Second, both groups were joined by two linguists in a workshop to define a final version, which was back-translated to French by the linguists and compared to the original French version. Lastly, the translation groups scored each item of the final HDRS-21 Kinyarwanda version compared with the original French version, scoring for cultural and linguistic clarity. The quality of the translation was measured by the inter-rater agreement on the assessment of the linguistic and cultural clarity. Agreement was calculated for all 21 questions using Cohen’s kappa coefficient (Cohen, 1960; McHugh, 2012).

### Validation of the Translated HDRS-21

#### Study Set-up

Data collection was conducted in Rwanda. This was a comparative study on a sample of patients living with depression and of healthy volunteers. Data analysis was conducted at Duke University, Durham, United States in 2017. The study was approved by the Institutional Review Board of the Kigali University Hospital, Rwanda, and participants provided written informed consent.

#### Study Participants

Individuals with depression were recruited from six healthcare and support centers in Rwanda: Neuropsychiatric Hospital Ndera, CARAES Neuropsychiatric Center in Butare, TRAC (research and treatment center for HIV/AIDS), Association Rwandaise des Conseillers en Traumatisme (ARCT, counseling organization for trauma), Uyisenga N’Manzi center (community center for orphaned children to young adults after the genocide), and the Association des Veuves de la Genocide (counseling organization for widows following the genocide).

Individuals attending these centers were invited to participate in the study if they were aged ≥18 years and were able to communicate clearly. Patients with depression were selected based on their medical charts, and if diagnosed with depression according to the fourth revision of the American Psychiatric Association’s Diagnostic and Statistical Manual (DSM-IV) or the tenth revision of the International Classification of Diseases and Related Health Problems (ICD-10) criteria by a structured HCP-conducted interview.

The control group consisted of healthy volunteers recruited at various sites (hospital, university, and health education institute).

#### Sample Size Calculation

Sample size calculation was estimated using Gorsuch’s rule (MacCallum et al., 1999), which requires a sample size of five times the number of questions assessed, a total of 21 in this study, resulting in sample sizes of 105 per group. Given that the total score only concerns the first 17 questions, we assumed this to be an adequate sample size.

#### Procedure

The HDRS-21 in Kinyarwanda was administered through a semi-structured clinical interview conducted by trained nurses and psychologists, trained general practitioners, and trained psychiatrists. Severity of depression was based on expert assessment by the lead investigator, a trained psychiatrist.

#### Psychometric Parameters and Statistical Analysis

All analyses were conducted with R Language for Statistical Computing (R foundation, Vienna, Austria). Missing data was imputed for cases where individual questions were not answered, up to three missing item responses. Generated by the mice package in the R Language for Statistical Computing, we used 15 multiple imputation sets, with the other questions in the questionnaire and sociodemographic variables as the sources for imputation.

##### Internal Structure

Confirmatory factor analysis (CFA) was used to test the internal structure of the HDRS. Testing was based on a unidimensional model, indicating that the score based on all items of the HDRS referred to a single construct, depression. Given the categorical nature of the data, the weighted least square means and variance-adjusted (WLSMV) estimator was used to estimate model parameters. The relationship between each item and depression was determined by its loading, which corresponds to the correlation between the item and depression.

Through the CFA analysis, several indices that provide a measure of the goodness-of-fit of the models to the data were obtained. The fit indices, and the generally accepted reference levels for a good fit (in parentheses), were the following: chi-square (*X*^2^ and *p*-value), root mean square error of approximation (RMSEA, ≤0.08), Tucker-Lewis index (TLI, >0.90), comparative fit index (CFI, ≥0.90), and average variance extracted (AVE, >0.50) (Hu and Bentler, 1999; Hair et al., 2005; Kline, 2011).

##### Reliability

To determine the reliability of the HDRS, its internal consistency was evaluated. Internal consistency, which indicates the degree to which all items in the instrument refer to the same construct, can be assessed by several coefficients, each with its own strengths and limitations (De Vellis, 2003; Padilla and Divers, 2016). For this study, Cronbach’s alpha, composite reliability (CR), and McDonald’s omega coefficient were calculated using CFA results.

##### Validity

The validity of the HDRS as a diagnostic tool was examined by evaluating its accuracy in distinguishing between levels of depression, in relation to the gold standard expert opinion. Sensitivity, specificity, positive predictive values, and negative predictive values of the tool were calculated. By plotting sensitivity versus specificity across the HDRS scores, receiver operator characteristic (ROC) curves were generated and the area under the ROC curve (AUC) was calculated. The AUC provides a summary measure of the sensitivity and specificity of the HDRS, relative to the expert opinion diagnostic, across the entire range of scores. The optimal cut-off score for maximum discrimination between depressed and non-depressed participants was determined from the ROC curves using the Youden index.

## Results

### Translation

The mean Cohen’s kappa coefficient of the composite score of all 21 items was 0.93, indicating a high degree of agreement between translators when comparing translated and original versions.

### Study Participants

Overall, 210 adults participated in the study, divided into two groups of 105 participants (Table 1). Of the depressed group, 26 (24.8%) were recruited at the Neuropsychiatric Hospital Ndera, 5 (4.8%) at CARAES Neuropsychiatric Center in Butare, 14 (13.3%) at TRAC, 5 (4.8%) at ARCT, 10 (9.5%) at Uyisenga N’Manzi center, and 45 (42.9%) at the Association des Veuves de la Genocide.

**Table 1:** Depression severity as per expert opinion.

	Patient group (*n* = 105)	Control group (*n* = 105)
Depression severity, *n* (%)		
No depression	0 (0%)	105 (100%)
Mild	14 (13.3%)	0 (0%)
Moderate	0 (0%)	0 (0%)
Severe	91 (86.6%)	0 (0%)

### Internal Structure

**Figure 1:**
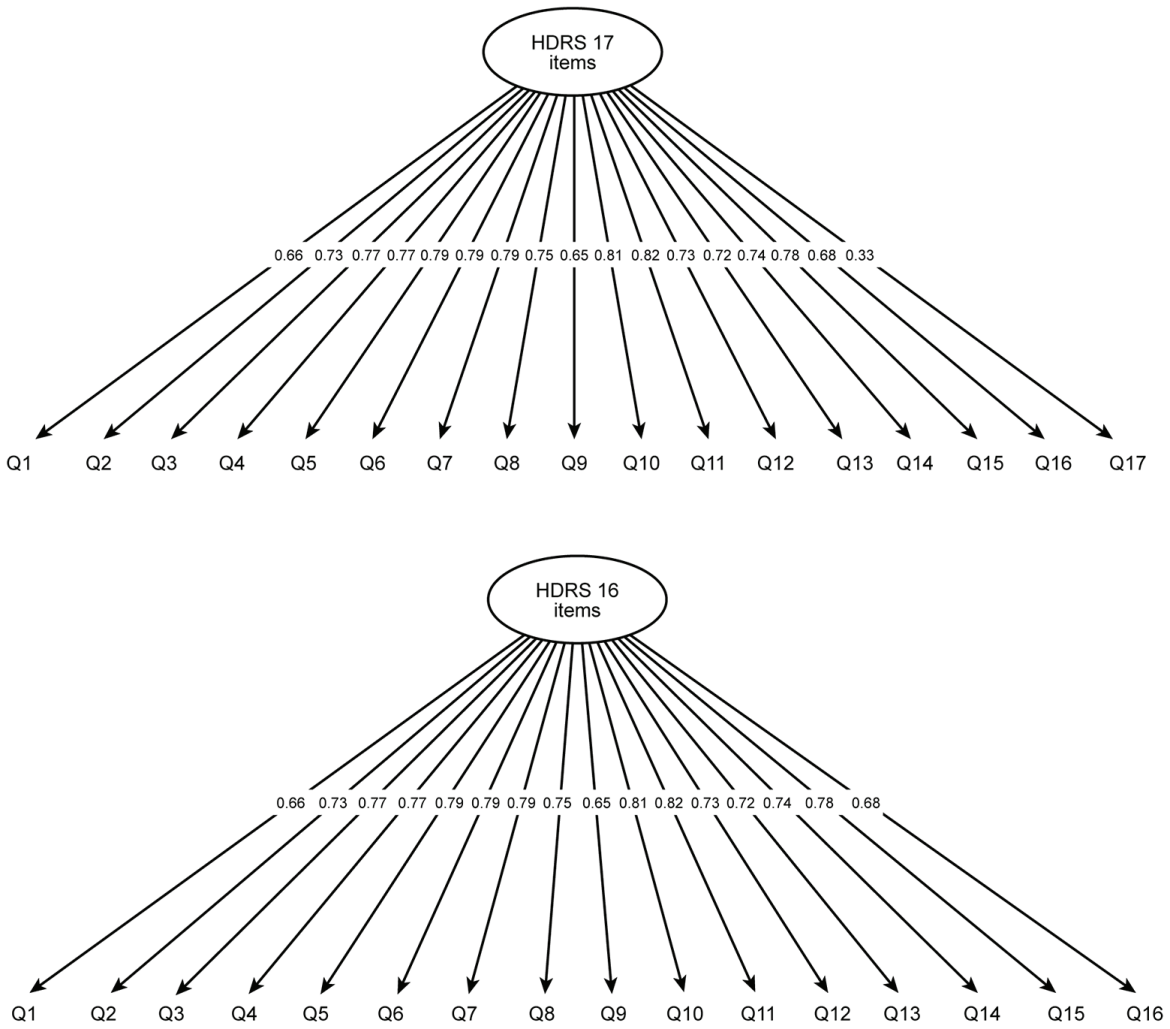
Confirmatory factor analysis model for the Hamilton Depression Rating Scale (HDRS) with factor loadings for 17 **(top)** and 16 items **(bottom)**. HDRS, Hamilton Depression Rating Scale.

The CFA unidimensional model performed well, showing all items with factor loadings ranging from 0.32 to 0.81 for the HDRS 17 (Figure 1). Item 17 of the HDRS (patient insight into his/her condition) showed the smallest factor loading, suggesting a problem with the item’s performance. Another model that excluded item 17 was tested and was also shown to perform well, with factor loadings ranging from 0.65 to 0.81 (Figure 1 and Table 2).

**Table 2:** Goodness-of-fit indices from the confirmatory factor analysis and reliability coefficients.

	HDRS 16 items	HDRS 17 items
Confirmatory factor analysis		
*X*^2^ (degrees of freedom); *p*-value	147.10 (104); *p* = 0.01	159.03 (119); *p* = 0.01
RMSEA (95% CI)	0.05 (0.03, 0.06)	0.04 (0.02, 0.06)
Tucker-Lewis index	0.99	0.99
Comparative fit index	0.99	0.99
Average variance extracted	0.56	0.53
Factor loading range	0.65–0.81	0.32–0.81
Reliability		
Cronbach’s alpha (95% CI)	0.92 (0.91, 0.93)	0.92 (0.90, 0.93)
Composite reliability	0.95	0.95
McDonald’s omega	0.93	0.93

CI, confidence interval; HDRS, Hamilton Depression Rating Scale; RMSEA, root mean square error of approximation.

Further results of the CFA confirmed that the models show a good fit to the data; the goodness-of-fit indices all met the criteria standards for adequacy of fit (Table 2).

### Reliability

Values obtained for all three reliability coefficients were above 0.80 for both HDRS 16 and 17 items, indicating strong internal consistency (Table 2).

### Validity

**Figure 2:**
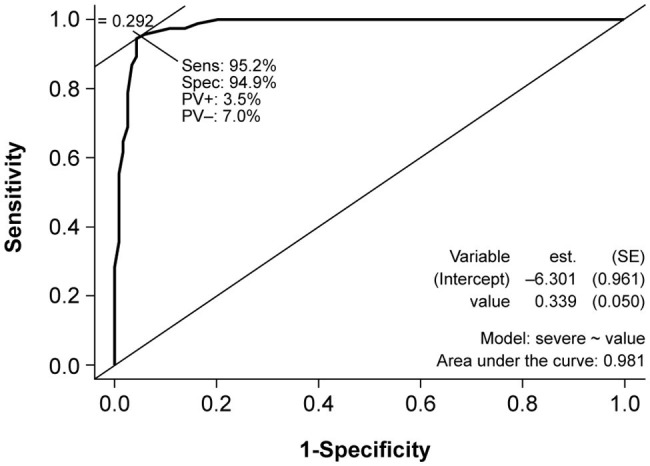
Receiver operator characteristic (ROC) curve for the diagnostic ability of the HDRS versus the depression diagnostic classification of each participant. est., estimate; HDRS, Hamilton Depression Rating Scale; PV, predictive value; ROC, receiver operator characteristic; SE, standard error; Sens, sensitivity; Spec, specificity.

The translated HDRS was associated with a high sensitivity (0.95) and specificity (0.94) in differentiating participants in the control group who did not have depression from those in the patient group who had moderate to severe depression (Figure 2).

At a cut-off point of 17 for diagnosis of depression, sensitivity and specificity were both 0.95 relative to gold standard. Positive and negative predictive values were 96.5 and 93% respectively, and the AUC was 0.98.

## Discussion

This study evaluated the psychometric properties of the HDRS-21 in Kinyarwanda and established the optimal cut-off for depression at a score of ≥17.

The translation process, using a mixed model including specialized HCPs and bilingual experts with multiple iterations, allowed us to address cultural sensitivities on depression and words describing feeling states. High consistency between the French and Kinyarwandan versions was reflected by Cohen’s kappa >0.8 and demonstrated excellent inter-group consistency of all translated items.

Subsequently, the psychometric properties of the translated HDRS were evaluated. Results of the various tests conducted to assess these properties support the use of the HDRS as a reliable and valid instrument for evaluating depression. Using CFA, the internal structure of the HDRS was found to be adequate in relation to a single construct, depression, as reported in other studies (Williams, 2001; Ruhe et al., 2005; Trajkovic et al., 2011). For a unidimensional model, all measuring items should have acceptable factor loadings. In our analysis, item 17 was associated with a smaller factor loading relative to the other 16 items, suggesting a poorer performance.

Reliability is the ability of an instrument to measure a given construct consistently. The most widely used method for evaluating the reliability of an instrument is Cronbach’s alpha (Tavakol and Dennick, 2011). As a sensitivity analysis, composite reliability and McDonald’s omega were also calculated. The strong internal consistency of the translated HDRS, with reliability coefficients >0.80, suggests that it is a highly reliable tool for assessing depression.

In addition to reliability, another fundamental element in the evaluation of a measurement instrument is its validity (Tavakol and Dennick, 2011). The validity of the translated HDRS was demonstrated by the AUC of 0.98, close to a value of 1.0 that indicates a perfectly accurate instrument.

The present study aimed to establish a diagnostic cut-off for the HDRS in Kinyarwanda. Based on the Youden index, a cut-off point of 17 was deemed optimal with sensitivity and specificity >95%. This cut-off for diagnosis is in line with similar cut-offs of ≥16 and 18/19 observed in a mixed community/outpatient sample (Mottram et al., 2000) and in a Parkinson’s disease outpatient setting in Ecuador, respectively (Serrano-Duenas and Soledad Serrano, 2008). In disease-specific populations, lower cut-offs have been observed for Parkinson’s disease (Leentjens et al., 2000) (≥12 and 15/16) (Naarding et al., 2002), stroke (10/11) (Naarding et al., 2002), and Alzheimer’s disease (13/14) (Naarding et al., 2002). In our study, only 11 patients had mild depression, skewing diagnosis to moderate to severe depression, which may have resulted in a high HDRS cut-off. This could be an advantage in settings with limited availability of drugs and where priorities for medical treatment need to be set toward moderate to severe depression. For example, in the treatment of epilepsy, the treatment of depression may not be suitable for patients with minor or mild depression (Rathore et al., 2014).

The main limitation of the study was the patient sample enrolled. First, the sample size according to Gorsuch’s rule allowed validation and assessment of its psychometric properties, but larger sample sizes have been proposed as well (MacCallum et al., 1999). This may explain the decreased factor loading of item 17, with a supplementary analysis using a 16-item model excluding item 17 also showing to be a good fit to the data. Until larger studies confirm our findings and validate a 16-item HDRS total score, we recommend to maintain the 17-item HDRS total score. Second, we recruited at six different sites, yet patients with a background of war trauma represented more than half of our sample, which may not have been representative of the general population. HDRS cut-offs also vary by concomitant disease or background and require confirmation if used in specific populations. Lastly, our study design did not allow for testing of inter‐ and intra-rater reliability. These limitations warrant future studies in a larger sample of the general population, including intraclass correlation as well as confirmation of the observed factor loading for item 17.

Evaluating psychometric properties of screening and assessment tools is a necessary process, since even minor issues in wording, connotation, and question structure can reduce the cross-cultural relevance of the tools (Sweetland et al., 2014). This is particularly important since use of these scales by trained nurses and other non-specialist staff is pivotal for scaling up mental health care in Rwanda and to a broader extent, in low-income countries, it increases the number of staff that can provide such care (Belkin et al., 2011; Sweetland et al., 2014). In 2012, the Rwandan Ministry of Health, in collaboration with Partners in Health, adapted a program for primary care nurses providing training to participate in the clinical care of people with mental disorders (Smith et al., 2017). In this stepped-up pathway, trained staff can participate more and more in screening and triage, engagement, follow-up, and monitoring, as well as referral when and where required (Sweetland et al., 2014). Consequently, availability of HDRS in Kinyarwanda is an important step forward given the high prevalence of depression.

The HDRS is a valuable addition to the armamentarium of validated tools in Kinyarwanda such as the Patient Health Questionnaire (PHQ)-9, validated for screening for depression, and the Center for Epidemiological Studies Depression Scale for Children (CES-DC) (Betancourt et al., 2012), addressing a vulnerable population. Although HDRS has also been used for screening for depression (Aben et al., 2002; Costa et al., 2016), we believe that the high number of questions and duration of administration impair its user-friendliness as a screening tool, for which PHQ-9 is available as a validated alternative.

Addressing the recommendation in the Rwandan Mental Health survey, the validation of the HDRS in Kinyarwanda (the Kinyarwanda version of HDRS can be accessed online here: http://caraesnderahospital.rw/research-2/) provides primary and secondary healthcare staff with an accurate tool for diagnosis of moderate to severe depression, enabling closure of the diagnosis and treatment gap.

## Data Availability Statement

The datasets presented in this article are not readily available due to patient confidentiality. Requests to access the datasets should be directed to peter.dedeken@heilighartlier.be.

## Ethics Statement

The studies involving human participants were reviewed and approved by Institutional Review Board of the Kigali University Hospital, Rwanda. The patients/participants provided their written informed consent to participate in this study.

## Author Contributions

FS and ER performed data collection. JV performed statistical analysis. JV, PD, DT, and PB developed concept of analysis. JV and PD co-authored the manuscript. FS, ER, JV, DT, PB, and PD reviewed and approved the manuscript. All authors contributed to the article and approved the submitted version.

### Conflict of Interest

PD has received consultancy fees from UCB Pharma and Novartis. DT is an employee of UCB Pharma. PB has received speaker fees and grants from several pharmaceutical companies, outside the scope of this study.

The remaining authors declare that the research was conducted in the absence of any commercial or financial relationships that could be construed as a potential conflict of interest.
